# Exploiting transplastomically modified Rubisco to rapidly measure natural diversity in its carbon isotope discrimination using tuneable diode laser spectroscopy

**DOI:** 10.1093/jxb/eru036

**Published:** 2014-03-31

**Authors:** Susanne von Caemmerer, Youshi Tazoe, John R. Evans, Spencer M. Whitney

**Affiliations:** Research School of Biology, The Australian National University, Canberra ACT 0200, Australia

**Keywords:** C_4_ photosynthesis, carbon isotope discrimination, *Flaveria*, Rubisco, tobacco, tuneable diode laser spectroscopy.

## Abstract

Carbon isotope discrimination (Δ) during C_3_ photosynthesis is dominated by the fractionation occurring during CO_2_-fixation by the enzyme Rubisco. While knowing the fractionation by enzymes is pivotal to fully understanding plant carbon metabolism, little is known about variation in the discrimination factor of Rubisco (*b*) as it is difficult to measure using existing *in vitro* methodologies. Tuneable diode laser absorption spectroscopy has improved the ability to make rapid measurements of Δ concurrently with photosynthetic gas exchange. This study used this technique to estimate *b in vivo* in five tobacco (*Nicotiana tabacum* L. cv Petit Havana [N,N]) genotypes expressing alternative Rubisco isoforms. For transplastomic tobacco producing *Rhodospirillum rubrum* Rubisco *b* was 23.8±0.7‰, while Rubisco containing the large subunit Leu-335-Val mutation had a *b*-value of 13.9±0.7‰. These values were significantly less than that for Rubisco from wild-type tobacco (*b*=29‰), a C_3_ species. Transplastomic tobacco producing chimeric Rubisco comprising tobacco Rubisco small subunits and the catalytic large subunits from either the C_4_ species *Flaveria bidentis* or the C_3_-C_4_ species *Flaveria floridana* had *b*-values of 27.8±0.8 and 28.6±0.6‰, respectively. These values were not significantly different from tobacco Rubisco.

## Introduction

Carbon isotope discrimination occurring during C_3_ photosynthesis is determined by CO_2_-diffusion processes from the atmosphere to the chloroplast and the biochemical fractionation occurring during CO_2_ fixation by Rubisco and during respiratory and photorespiratory CO_2_ release (Farquhar *et al.*, 1989a). The fact that Rubisco discriminates strongly against ^13^CO_2_ is apparent in the isotopic signature of atmospheric CO_2_ and this has become a tool for monitoring global CO_2_ exchange processes (Mook *et al.*, 1983; Yakir and Sternberg, 2000). The strong ^13^CO_2_ discrimination by Rubisco is the primary cause of depleted ^13^C levels in plant biomass. This effect has proved experimentally versatile by allowing photosynthetic carbon isotope discrimination to be used as a tool to elucidate CO_2_-diffusion processes through stomata and from the leaf intercellular airspace to the sites of Rubisco carboxylation in the chloroplast stroma of C_3_ plant species (Evans *et al.*, 1986, 2009; Farquhar *et al.*, 1989b). Interpreting ^13^CO_2_ discrimination in C_4_ plants has proved more challenging as a CO_2_-concentrating mechanism (CCM) operates that spatially localizes Rubisco in bundle sheath compartments with reduced access to atmospheric CO_2_. In the C_4_ photosynthetic CCM, initial fixation of atmospheric CO_2_ occurs via phosphoenolpyruvate carboxylase (PEPC), which discriminates less against ^13^C than Rubisco (Farquhar, 1983). C_4_ acids diffuse into the bundle sheath where decarboxylation supplies CO_2_ to Rubisco. As a result of this CCM pathway, photosynthetic carbon isotope discrimination is much less in C_4_-plant species (Evans *et al.*, 1986; Henderson *et al.*, 1992).

The fractionation factor of Rubisco is difficult to measure and only a limited number of measurements exist (McNevin *et al.*, 2007 and references therein). Current methods rely on the purification of natural or recombinant Rubisco forms by processes that typically reduce catalytic activity (Sharwood *et al.*, 2008). In plants, algae, and cyanobacteria, Rubisco is a 520–550-kDa L_8_S_8_ hexadecamer composed of eight ~50-kDa catalytic large (L) subunits and eight ~12–15-kDa small (S) subunits (Whitney *et al.*, 2011a). In most applications of photosynthetic carbon isotope discrimination, the fractionation factor of plant L_8_S_8_ Rubisco is assumed to be ~29‰, a value reproducibly derived for spinach Rubisco *in vitro* using a range of experimentally complex methodologies (Roeske and O’Leary, 1984) and supported by *in vivo* measurements of carbon isotope discrimination in transgenic tobacco with reduced amounts of Rubisco (Evans *et al.*, 1994). However, the evolutionary diversity in Rubisco catalysis (Yeoh *et al.*, 1981; Badger and Andrews, 1987; Tcherkez *et al.*, 2006), even among closely related C_3_ species (Delgado *et al.*, 1995; Galmes *et al.*, 2005), brings into question the validity of this assumption. This catalytic diversity may conceivably arise from subtle variations to the reaction mechanism of Rubisco. Differences in the fractionation factor of Rubisco pose a useful means for interpreting such reaction mechanism variations (Tcherkez *et al.*, 2006; McNevin *et al.*, 2007; Tcherkez, 2013).

Transgenic tobaccos with altered amounts or forms of Rubisco have been used to quantify the enzyme’s kinetic properties using leaf gas exchange and photosynthesis models. This *in vivo* approach has been particularly successful in determining the Michaelis–Menten constants for CO_2_ and O_2_ (*K*_c_ and *K*_o_), catalytic turnover rates (*V*_Cmax_ and *V*_Omax_) and CO_2_/O_2_ specificity of tobacco Rubisco and how they vary with temperature (von Caemmerer *et al.*, 1994; Bernacchi *et al.*, 2002; Walker *et al.*, 2013). The approach has also been successfully applied to catalytically altered Rubisco isoforms expressed in tobacco using chloroplast transformation technology (Whitney *et al.*, 1999; Whitney and Andrews, 2003; Sharwood *et al.*, 2008). More recent developments in tuneable diode laser (TDL) absorption spectroscopy have improved the ability to make rapid measurements of carbon isotope discrimination concurrently with photosynthetic gas exchange (Tazoe *et al.*, 2011). The current study combines this technique with transplastomic tobacco lines expressing alternative Rubisco isoforms to measure the Rubisco discrimination factor *in vivo*. The results confirm the fractionation factors determined *in vitro* for Rubisco from *Rhodospirillum rubrum* and the mutant tobacco Leu-335-Val (L335V) Rubisco (McNevin *et al.*, 2007) and also show that Rubisco fractionation factors for Rubisco from *Flaveria bidentis* (a C_4_ species) and *Flaveria floridana* (C_3_-C_4_ intermediate species) are similar to that from tobacco (a C_3_ species).

## Materials and methods

### Plant material

This study used wild-type tobacco (tob(Wt), *Nicotiana tabacum* L. cv Petit Havana [N,N]) and transplastomic mutants producing *R. rubrum* Rubisco (tob(Rr), Whitney and Andrews, 2001), mutant tobacco Rubisco containing the large subunit Leu-335-Val substitution (tob(L335V), Whitney *et al.*, 1999), or hybrid Rubisco comprising tobacco small subunits and *F. bidentis* (tob(bid), Whitney *et al.*, 2011b) or *F. floridana* (tob(flo), Whitney *et al.*, 2011b) large subunits. As some of the transplastomic mutants could not grow in air, all plants were grown in a growth chamber supplemented with 1% (v/v) CO_2_. The air temperature was 25 °C with a 14-h photoperiod (400 μmol photon m^–2^ s^–1^) and 60% relative humidity.

### Concurrent gas exchange and carbon isotope discrimination measurements

Gas exchange and carbon isotope discrimination measurements were made as described by Tazoe *et al.* (2011) using either a 6-cm^2^ chamber of the LI-6400 with a red-blue light-emitting diode (LED) light source (Li-Cor, Lincoln, Nebraska, USA) or a laboratory-constructed whole-leaf chamber (115×110×25mm depth, boundary layer conductance 4mol m^–2^ s^–1^) together with a red-green-blue LED light source (6400–18 RGB Light source, Li-Cor) and the LI-6400. The flow rate was set at 200 μmol s^–1^. Gas exchange was coupled to a tuneable diode laser (TDL, TGA100, Campbell Scientific, Logan, UT, USA) for concurrent measurements of carbon isotope composition. Measurements were made at 4-min intervals for 20 s and between six and eight measurements were made at each CO_2_ partial pressure at an irradiance of 1500 μmol quanta m^–2^ s^–1^. Other measurement conditions were O_2_ 19 mbar, and a leaf temperature 25 ºC. The LI-6400 CO_2_ mixing system was used to generate different CO_2_ concentrations. The δ^13^C of CO_2_ gas cylinders (δ^13^ *C*_tank_) used in the LI-6400 CO_2_ injector system was between –13 and –3‰. Gas exchange was calculated using the equations presented by von Caemmerer and Farquhar (1981) and Δ was calculated from the equation presented by Evans *et al.* (1986) as:

$$\Delta =\frac{1000\xi \left(\delta^{13}C_{sam}-\delta^{13}C_{ref}\right)}{1000+\delta^{13}C_{sam}-\xi \left(\delta^{13}C_{sam}-\delta^{13}C_{ref}\right)}$$ (1)

where δ^13^C_sam_ and δ^13^C_ref_ are the carbon isotope compositions of the leaf chamber and reference air of the LI-6400, respectively, ξ is *C*_ref_/(*C*_ref_–*C*_sam_), where *C*_ref_ and *C*_sam_ are the CO_2_ concentrations of dry air entering and exiting the leaf chamber, respectively, measured by the TDL. The value of ξ ranged from 4.5 to 13 for tob(Wt), 15 to 25 for tob(L335V), 15 to 16 for tob(Rr), 11 to 16 for tob(bid), and 8 to 15 for tob(flor).

### Biochemical measurements

Following gas exchange, replicate leaf samples (0.5cm^2^) were taken from the sampling area and immediately frozen in liquid nitrogen and stored at –80 °C. Rubisco content in each sample was measured by the [^14^C]carboxyarabinitol–P_2_-binding assay procedure according to Ruuska *et al.* (1998). Soluble leaf protein was measured relative to BSA with a dye-binding assay (Pierce Coomassie Plus Kit). Dry mass of leaves were measured after 48h at 80 °C. Leaf dry mass per unit area was calculated from destructive harvest data taken from 10 plants after 34 d.

Rubisco kinetic properties of Rubisco in tob(Rr) leaf protein extract was measured at 25 °C using ^14^CO_2_-fixation assays as described (Whitney and Sharwood, 2007; Sharwood *et al.*, 2008). Assays were performed in 8-ml septum capped vials containing 1ml reaction buffer [50mM HEPES-NaOH pH 7.8, 15mM MgCl_2_, 0.25mM ribulose bisphosphate (RuBP)] and varying concentrations of NaH^14^CO_3_ (9–952 μM) and O_2_ (0, 10, 15 and 20% (v/v), accurately mixed with nitrogen using Wosthoff gas mixing pumps). Leaf protein was extracted in activation buffer [50mM HEPES-NaOH pH 7.8, 15mM MgCl_2_, 20mM NaH^14^CO_3_, 0.5mM EDTA, 2mM dithiothreitol, 1%, v/v, plant protease inhibitor cocktail (Sigma-Aldrich), and 1%, w/v, polyvinylpolypyrrolidone] and the Rubisco was activated at 25 °C for 10min prior to using 20 μl to initiate the assays. The Michaelis constants (*K*_*m*_) for CO_2_ (*K*_c_) and O_2_ (*K*_o_) were determined from the fitted data. The maximal carboxylation rate extrapolated from Michaelis–Menten curve fitting was divided by the amount of Rubisco active sites quantified by [^14^C]carboxyarabinitol-P_2_ binding (Ruuska *et al.*, 1998; Whitney and Andrews, 2001) to give k_ccat_.

### Calculation of Rubisco fractionation and mesophyll conductance

A full description of discrimination during C_3_ photosynthesis is given by Evans *et al.* (1986). However, Farquhar and Cernusak (2012) pointed out that while equations used to calculate gas exchange include ternary effects of transpiration rate on the rate of CO_2_ assimilation through stomata (von Caemmerer and Farquhar, 1981), the equations describing carbon isotope discrimination had been derived without the ternary effects. They introduced revised equations, and these are used in the current calculation:

$$\begin{array}{l} \Delta =\frac{1}{1-t}a^{\prime }+\frac{1}{1-t}\left(\left(1+t\right)b-a^{\prime }\right)\frac{C_{i}}{C_{a}}-\frac{1+t}{1-t}\left(b-a_{i}-\frac{eR_{d}}{\left(A+R_{d}\right)}\right)\;\frac{A}{g_{m}C_{a}}\\ \;-\frac{1+t}{1-t}\left(\frac{eR_{d}}{(A+R_{d})C_{a}}\left(C_{i}-\Gamma_{*}\right)\right)-\frac{1+t}{1-t}\left(f\frac{\Gamma_{*}}{C_{a}}\right) \end{array}$$ (2)

where

$$t=\frac{\left(1+a^{\prime }\right)E}{2g_{ac}^{t}}$$

E denotes the transpiration rate, and $g_{\text{ac}}^{\text{t}}$ denotes the total conductance to CO_2_ diffusion including boundary layer and stomatal conductance (von Caemmerer and Farquhar, 1981). *C*_a_ and *C*_i_ are the ambient and intercellular CO_2_ partial pressures and Γ_*_ is the compensation point in the absence of mitochondrial respiration. *A* and *R*_d_ stand for CO_2_-assimilation rate and mitochondrial respiration in the light.

The mesophyll conductance to CO_2_ diffusion from intercellular airspace to the chloroplast, *g*_m_, is given by:

$$g_{m}=A/\left(C_{i}-C_{c}\right)$$ (3)

where *C*_c_ is the CO_2_ partial pressure in the chloroplast. The symbol *a*_i_ (1.8‰) denotes the fractionation factor for hydration and diffusion through water, and *b* (usually ~29‰) is the fractionation associated with Rubisco carboxylation. The symbol *a*′ denotes the combined fractionation factor through the leaf boundary layer and through stomata:

$$a^{\prime }=\frac{a_{b}\left(C_{a}-C_{s}\right)+a\left(C_{s}-C_{i}\right)}{\left(C_{a}-C_{i}\right)}$$ (4)

where C_s_ is the CO_2_ partial pressure at the leaf surface, *a*_b_ (2.9‰) is the fractionation occurring through diffusion in the boundary layer and *a* (4.4‰) is the fractionation due to diffusion in air (Evans *et al.*, 1986). The current study uses the photorespiratory fractionation factor *f* (16.2‰), determined by Evans and von Caemmerer (2013). Following Tazoe *et al.* (2009), no fractionation by day respiration is assumed and *e* is calculated as δ^13^ *C*_tank_–δ^13^ *C*_atmosphere_ (Wingate *et al.* 2007). In this study, δ^13^ *C*_tank_ ranged from –13.3 to –3‰ and δ^13^ *C*_atmosphere_ was –18‰ for plants grown in a growth cabinet with CO_2_ enrichment (McNevin *et al.*, 2007).

Evans and von Caemmerer (2013) solved equation 2 for *g*_m_, but this study has solved it for the Rubisco fractionation factor *b*:

$$b=\frac{\Delta -\frac{a^{\prime }}{1-t}\left(1-\frac{C_{i}}{C_{a}}\right)+\Delta_{e}+\Delta_{f}-\frac{1+t}{1-t}\left[\left(a_{i}+\frac{eR_{d}}{A+R_{d}}\right)\frac{A}{g_{m}C_{a}}\right]}{\frac{1+t}{1-t}\left(\frac{C_{i}}{C_{a}}-\frac{A}{g_{m}C_{a}}\right)}$$ (5)

where

$$\Delta_{\text{e}}=\frac{1+t}{1-t}\left(\frac{{\text{eR}}_{\text{d}}}{\left(\text{A}+{\text{R}}_{\text{d}}\right)C_{\text{a}}}\left(C_{\text{i}}-\Gamma_{\text{*}}\right)\right)$$ (6)

is most of the fractionation associated with respiration and

$$\Delta_{f}=\frac{1+t}{1-t}\left(f\frac{\Gamma_{*}}{C_{a}}\right)$$ (7)

is the fractionation associated with photorespiration.

## Results

### Gas exchange and biochemical properties of tobacco genotypes

This study used five tobacco (*N. tabacum* L. cv Petit Havana [N,N]) genotypes: wild-type [tob(Wt)] and transplastomic mutants producing homodimeric L_2_ *R. rubrum* Rubisco [tob(Rr)], Whitney and Andrews, 2001; tobacco Rubisco containing the L-subunit Leu-335-Val mutation [tob(L335V)], Whitney *et al.*, 1999, or producing chimeric L_8_S_8_ Rubisco comprising tobacco S-subunits and either the *F. bidentis* L-subunit [tob(bid)], Whitney *et al.*, 2011b or the *F. floridana* L-subunit [tob(flo)], Whitney *et al.*, 2011b]. Table 1 summarizes *in vitro* catalytic properties of these enzymes and compares them to the catalytic properties of the native enzyme.

**Table 1.** *In vitro* Rubisco kinetic constants of wild-type tobacco and *Flaveria floridana*, *Flaveria bidentis*, *Rhodospirillum rubrum*, and transplastomic mutants tob(flo), tob(bid), tob(Rr), and tob(L335V)To convert values from concentrations to partial pressures, solubilities for CO_2_ of 0.0334mol (l bar)^–1^ and for O_2_ of 0.00126mol (l bar)^–1^ were used. Atmospheric pressure in Canberra has an average of 953 mbar.

Rubisco type	*S*_c/o_ (MM^–1^)	*S*_c/o_ (bar bar^–1^)	*k*_ccat_ (s^–1^)	*K*_c_ (μM)	*K*_c_ (μbar)	k_ocat_ (s^–1^)	*K*_o_ (μM)	*K*_o_ (mbar)	Reference
Tobacco	81±1	2147±27	3.2±0.2	12.6±0.2	377±6	0.8	274±18	217±14	Whitney *et al.* (2011)
*F. floridana*	82±2	2174±53	3.6±0.1	14.4±0.5	431±15	1.1	374±33	297±26	
tob(flo)	81±2	2147±53	3.7±0.2	14.5±0.3	434±9	1.2	359±22	285±17	
*F. bidentis*	81±1	2147±27	4.8±0.3	20.4±0.5	611±15	1.2	420±37	333±29	
tob(bid)	79±2	2094±53	4.7±0.2	19.9±0.6	596±18	1.2	408±28	324±22	
*R. rubrum*	9±0.3	239±8	12.3±0.3	149±8	4461±240	1.4	159±25	126±19	Mueller-Cajar *et al.* (2007)
tob(Rr)	12±1	318±27	5.4±0.3	96±5	2874±150	0.34	72±9	57±7	This study
tob(L335V)	20±2	530±53	0.8±0.1	5.1±0.8	153±24	0.4	49±11	38.9±8.7	Whitney *et al.* (1999)

**Fig. 1.**
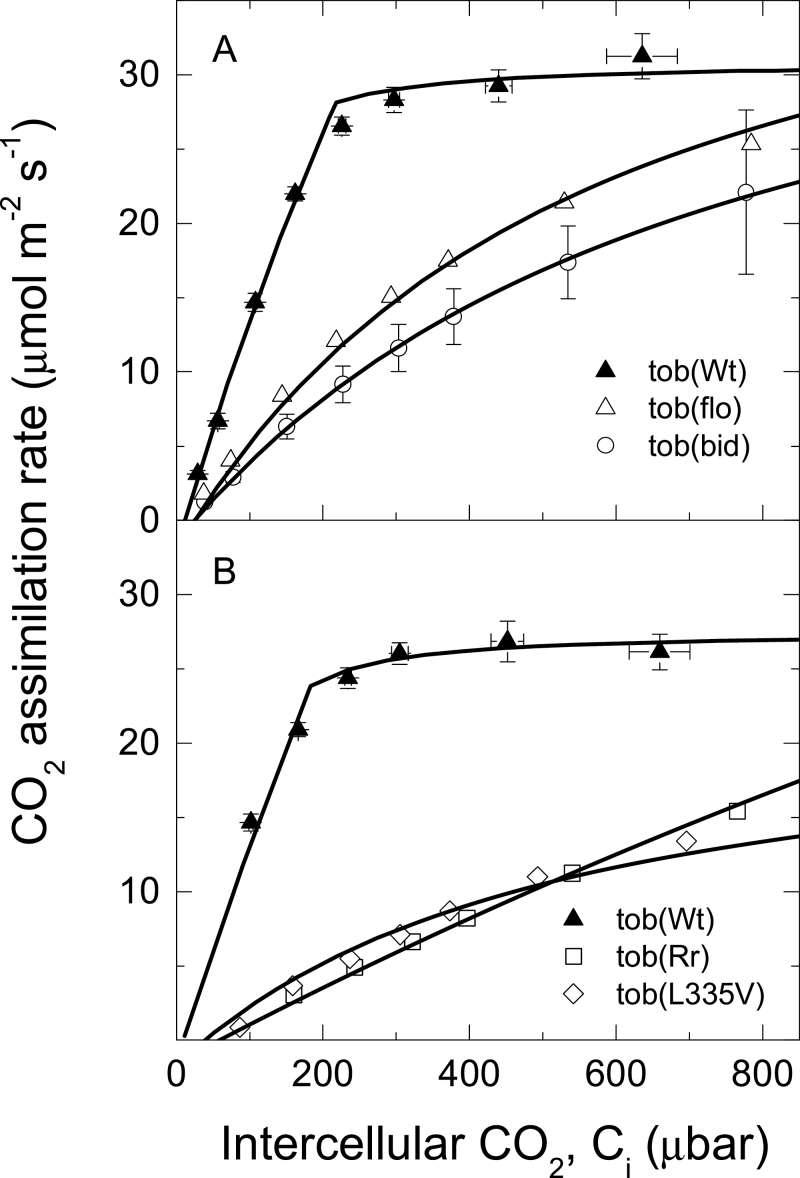
(A) CO_2_-assimilation rate, *A*, as a function of intercellular CO_2_ partial pressure in tobacco wild type [tob(wt)] and two transplastomic mutants producing large subunits of *Flaveria bidentis* [tob(bid)] or *F. floridana* [tob(flo)]. Measurements were made on four tob(wt), three tob(bid), and three tob(flo) replicate plants and bars show standard errors. (B) CO_2_-assimilation rate, *A*, as a function of intercellular CO_2_ partial pressure in tob(Wt) and two transplastomic mutants producing *R. rubrum* Rubisco [tob(Rr)] or tobacco mutant Rubisco [tob(L335V)]. Measurements were made on four tob(Wt), four tob(Rr), and seven tob(L335V) replicate plants and bars show standard errors. Gas exchange measurements were made at various CO_2_ partial pressures, O_2_ 19 mbar, irradiance 1500 μmol m^–2^ s^–1^, and leaf temperature 25 ºC. Model curves have been fitted to each genotype with the following values from Tables 1 and 2 [except tob(L335V); see text] and for *K*_c_ (μbar), *K*_o_ (mbar), Γ_*_ (μbar), *R*_d_ (μmol m^–2^ s^–1^) and *V*_cmax_ (μmol m^–2^ s^–1^). In A, for tob(Wt) 377, 217, 4.66, 1.8, 111.8, for tob(flo) 434, 285, 46.6, 1.9, 46.6, for tob(bid) 596, 324, 4.78, 1.3, 43.4, using *g*_m_ 0.46mol m^–2^ s^–1^ bar^–1^, and *J* 130.8 μmol m^–2^ s^–1^. In B, for tob(Wt) 377, 217, 4.66, 1.4,134.3, for tob(Rr) 2874, 57, 31.44, 0.8, 112.4, for tob(L335V) [in vivo constants used, see text] 318, 55.6, 140, 1.24, 23.4, using *g*_m_ 0.29mol m^–2^ s^–1^ bar^–1^, and *J* 115.6 μmol m^–2^ s^–1^.

All gas exchange measurements were made at low O_2_ partial pressure (19 mbar, ~2% atmospheric *p*O_2_) to ensure adequate CO_2_-assimilation rates could be measured at intercellular CO_2_ pressures between 100 and 800 μbar for all tobacco genotypes and to minimize photorespiratory fractionation. CO_2_ response curves of tob(Wt) show a clear transition from a Rubisco-limited to an RuBP-regeneration-limited response, whereas the other four genotypes remain Rubisco limited over the measured range in intercellular *p*CO_2_, with lower CO_2_-assimilation rates compared to wild type (Fig. 1). In tob(bid) and tob(flo) leaves, reduced CO_2_-assimilation rates were associated with a 2.5–4-fold lower Rubisco content in their leaves compared to wild type (Table 2 and Fig. 1A). Conversely, both tob(Rr) and tob(L335V) had slightly more Rubisco than wild type on a leaf area basis (Table 2), but the combination of lower *S*_c/o_ and reduced carboxylation efficiencies (*K*_ccat_/*K*_c_) resulted in CO_2_-assimilation rates that were still carboxylation limited at 800 μbar *p*CO_2_ and 19 mbar *p*O_2_ (Fig. 1B). Even under these low O_2_ conditions, both tob(L335V) and tob(Rr) have higher CO_2_ compensation points compared with tob(Wt), consistent with their significantly lower Rubisco CO_2_/O_2_ specificity (*S*_c/o_) and lower *K*_ccat_/*K*_c_ ratios (Table 1 and Fig. 1B). Although Rubisco from tob(bid) and tob(flo) share comparable *S*_c/o_ values with tob(Wt) (Table 1), their lower *K*_ccat_/*K*_c_ ratios increase their compensation points (Fig. 1A).

**Table 2.** Gas exchange and biochemical properties of wild-type tobacco and transplastomic mutants tob(Rr), tob(L335V), tob(bid), and tob(flo)Gas exchange and carbon isotope discrimination were measured at ambient CO_2_ ~380 μbar, O_2_ 19 mbar, irradiance 1500 μmol m^–2^ s^–1^, and leaf temperature 25 ºC. Other measurements were made on leaf material harvested from the same leaves after gas exchange measurements. ND, not determined.

Parameter	Set 1			Set 2		
	tob(Wt) (*n*=4)	tob(Rr) (*n*=4)	tob(L335V) (*n*=7)	tob(Wt) (*n*=4)	tob(bid) (*n*=3)	tob(flo) (*n*=3)
CO_2_-assimilation rate, *A* (μmol CO_2_ m^–2^ s^–1^)	26.0±0.8	6.6±0.2	7.1±0.3	30.2±0.9	13.4±1.9	17.0±0.4
Stomatal conductance (mol m^–2^ s^–1^)	0.57±0.08	0.57±0.04	0.31±0.04	0.64±0.06	0.52±0.07	0.74±0.07
Ratio of intercellular to ambient CO_2_, *C*_i_/*C*_a_	0.77±0.03	0.93±0.01	0.86±0.02	0.74±0.03	0.86±0.01	0.86±0.01
Dark respiration, *R*_d_(μmol CO_2_ m^–2^ s^–1^)	1.4±0.14	0.81±0.1	1.24±0.14	1.8±0.3	1.3±0.05	1.9±0.3
Mesophyll conductance, *g*_m_ (mol m^–2^ s^–1^ bar^–1^)	0.29±0.02	ND	ND	0.46±0.07	ND	ND
Rubisco sites (μmol CO_2_ m^–2^)	23.1±1.5	28.2±1.2	32.2±1.8	24.7±0.7	7.9±0.7	10.9±0.3
Maximum Rubisco activity, *V*_cmax_ (μmol CO_2_ m^–2^ s^–1^)^*a*^	134±6	112±2	23.8±1	116±6	43±7	44±2
Catalytic turnover of Rubisco *in vivo*, *k*_cat_ (s^–1^)^*b*^	5.9±0.3	4.1±0.1	0.75±0.03	4.7±0.2	5.4±0.2	4.1±0.3
Soluble protein (g m^–2^)	6.7±0.4	6.1±0.2	6.7±0.0.2	7.4±0.1	7.0±0.2	6.6±0.0.2
Leaf dry mass per unit leaf area (g m^–2^)	18.2±1.5	19.2±1.4	22.8±2.3	23.1±1.0	22.6±1.2	25.5±1.8

^*a*^ Maximum Rubisco activity, *V*_cmax_, was estimated from measurements of CO_2_ response curves using kinetic parameter values given in the legend of Fig. 1.

^*b*^ *k*_cat_ was calculated from the ratio of *V*_cmax_ and Rubisco site content measured on individual leaves.

Maximum Rubisco activity, *V*_cmax_, was estimated from CO_2_ response curves using the photosynthetic model of Farquhar et al. (1980). *In vitro* Rubisco kinetic constants *K*_c_, *K*_o_, and *S*_c/o_ given in Table 1 were used, with the exception of tob(L335V) where *in vivo* constants from Whitney *et al.* (1999) were used. CO_2_ partial pressures at the sites of carboxylation were calculated using the mesophyll conductance derived from wild-type tobacco grown at the same time (Table 2). Estimates of *in vivo k*_ccat_, calculated by dividing *V*_cmax_ by Rubisco site content per unit leaf area, assuming full activation, reflected *in vitro* variation in Table 1.

**Fig. 2.**
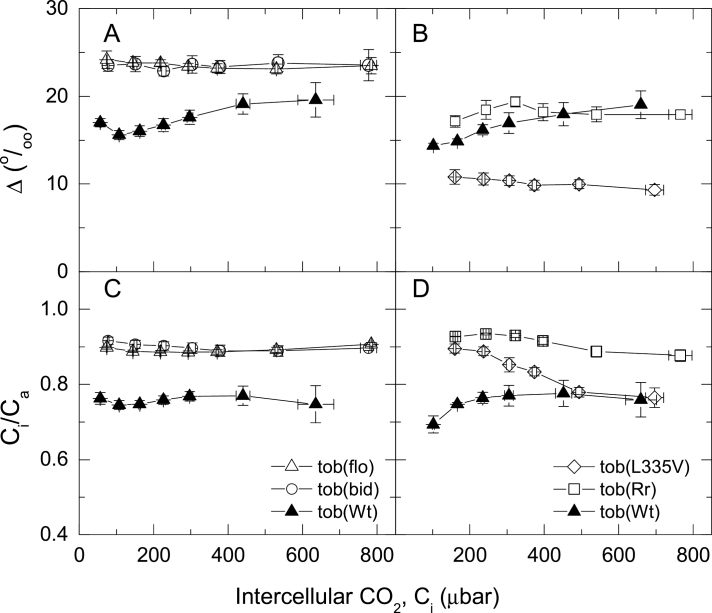
Carbon isotope discrimination measured concurrently with gas exchange (A and B) and the ratio of intercellular to ambient CO_2_, *C*_i_/*C*_a_ (C and D) in tobacco wild type [tob(Wt)] and transplastomic mutants. Transplastomic mutants and gas exchange details are as described for Fig. 1.

Stomatal conductance was relatively unchanged for the four tobacco mutants, despite having lower CO_2_-assimilation rates. Consequently, the mutants had greater ratios of intercellular to ambient CO_2_ (*C*_i_/*C*_a_; Table 2) for most of the *p*CO_2_ conditions tested (Fig. 2).

### Carbon isotope discrimination of tobacco genotypes

**Fig. 3.**
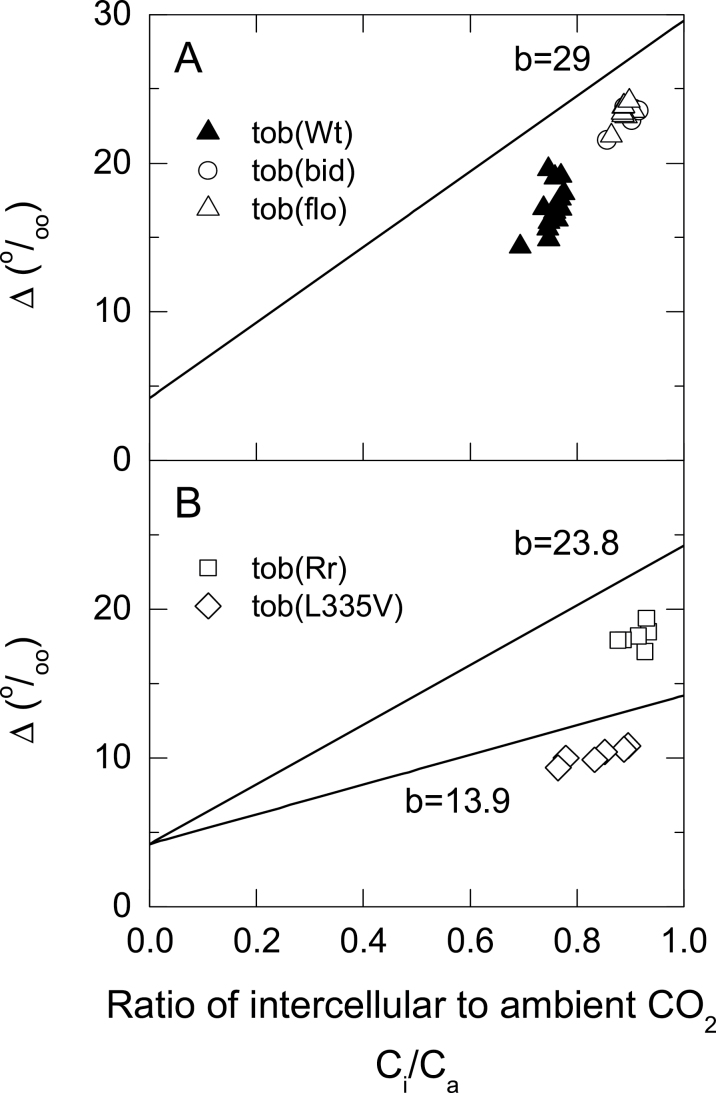
Carbon isotope discrimination, Δ, as a function of the ratio of intercellular to ambient CO_2_ partial pressure for tob(Wt), tob(bid), tob(flo), tob(Rr), and tob(L335V). Lines show theoretical relationships between Δ and *C*_i_/*C*_a_ with different Rubisco discrimination factors (*b*) which assume an infinite *g*_m_ and no respiratory fractionations, but include the ternary correction with *t*=0.01 ($\Delta =4.2+(1.02*b-4.2)C_{i}/C_{a}$ ). Transplastomic mutants are as described for Fig. 1.

This study measured the carbon isotope discrimination (Δ, ‰) concurrently with gas exchange using tuneable laser spectroscopy (Figs 2 and 3). The discrimination by both tob(bid) and tob(flo) was greater than that of tob(Wt) at all *p*CO_2_ (Fig. 2A). Under the range of *p*CO_2_ examined, carbon isotope discrimination by tob(L335V) was considerably less than tob(Wt) (Fig. 2B). In contrast, tob(Rr) had a greater discrimination at low *p*CO_2_ and became more similar to tob(Wt) at high *p*CO_2_. Discrimination is also shown against *C*_i_/*C*_a_ (Fig. 3) because discrimination is strongly influenced by *C*_i_/*C*_a_. *C*_i_/*C*_a_ was greater for all the mutants compared to tob(Wt) with the exception of tob(L335V) at high *p*CO_2_ (Fig. 2 C and D).

**Fig. 4.**
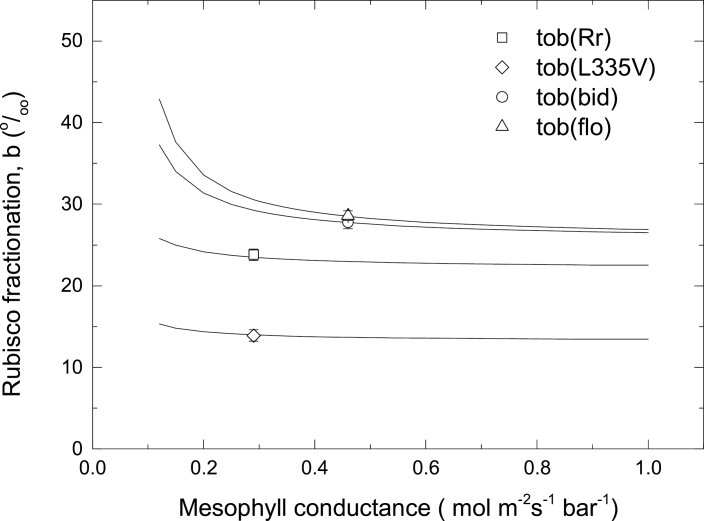
Modelled dependence of estimates of Rubisco fractionation factor, *b*, on mesophyll conductance using equation 5 and values of parameters given in Tables 2 and 3. Also shown are the measured values of *b* given in Table 2. Transplastomic mutants are as described for Fig. 1.

The average values of carbon isotope discrimination at ambient *p*CO_2_ are shown in Table 3. Prior studies of carbon isotope discrimination by tobacco showed that Rubisco fractionation (*b*) was independent of variation in mesophyll conductance, *g*_m_, and similar between the wild-type and anti-*Rbc*S plants which yielded an estimated value of *b*=29‰ (Evans *et al.*, 1994). Based on these observations, the current study assumed a value for *b*=29‰ for wild-type tobacco to estimate *g*_m_ and then calculated *b*-values for Rubisco from the four tobacco mutant genotypes using equation 5 by assuming the same *g*_m_ value to that measured in wild-type leaves of comparable physiological age and development (Table 3). This assumption is examined in Fig. 4, which shows that estimated *b*-values are relatively insensitive to changes in *g*_m_ until it is reduced below 50% of the assumed value, where *b* increases. If *g*_m_ in the transplastomic lines was 25% less than in wild-type leaves, estimated *b*-values would increase slightly to 24, 14.3, 28.6, and 29.6‰ for tob(Rr), tob(L335V), tob(bid), and tob(flo), respectively, which is within the margin of error for the values given in Table 3.

**Table 3.** Leaf carbon isotope discrimination and Rubisco discrimination (*b*) as well as carbon isotope discrimination associated with respiration (Δ_e_, equation 6) and photorespiration (Δ_f_, equation 7) in wild-type tobacco and transplastomic mutants tob(Rr), tob(L335V), tob(bid), and tob(flo)Gas exchange and carbon isotope discrimination were measured at ambient CO_2_ ~380 μbar, O_2_ 19 mbar, irradiance 1500 μmol m^–2^ s^–1^, and leaf temperature 25 ºC. To calculate Δ_f_, a value for Γ_*_ of 4.7 μbar was used for tob(Wt), tob(bid), and tob(flo), 14.0 μbar for tob(L335V), and 31.4 μbar for tob(Rr).

Parameter	Set 1			Set 2		
	tob(Wt) (*n*=4)	tob(Rr) (*n*=4)	tob(L335V) (*n*=7)	tob(Wt) (*n*=4)	tob(bid) (*n*=3)	tob(flo) (*n*=3)
Δ (‰)	16.9±1.2	19.4±0.6	10.4±0.6	16.9±0.6	21.6±0.9	21.8±0.3
Rubisco discrimination, *b* (‰)^*a*^	29	23.8±0.7	13.9±0.7	29	27.8±0.8	28.6±0.6
Rubisco discrimination (*b*) *in vitro* (McNevin *et al.* 2007) (‰)^b^	28.5±0.9	23.3±2.1	12.3±1.6			
Δ_e_ (‰)	0.2±0.01	0.5±0.06	1.2±0.2	0.6±0.1	1.2±0.2	1.1±0.3
Δ_f_ (‰)	0.1±0.001	1.4±0.02	0.6±0.003	0.2±0.001	0.2±0.001	0.2±0.0.001

^*a*^ Rubisco discrimination *b*, was estimated from Δ measured at ambient CO_2_ of 380 μbar using equation 5 and the *g*_m_ value of the wild-type control (Table 2).

^*b*^Expressed here with respect to gaseous CO_2_.

Respiratory and photorespiratory fractionations were calculated using equations 6 and 7 (Table 3). Although CO_2_-assimilation rates were lower in the four mutant tobacco genotypes (Fig. 1), the respiration rates were similar (Table 2). Consequently, the values of respiratory fractionation (Δ_e_) are slightly greater for the mutants compared to tob(Wt). Photorespiratory fractionation (Δ_f_) was greater in both tob(Rr) and tob(L335V) because these Rubiscos have lower *S*_c/o_ values which increases flux through photorespiration compared to tob(wt) (Table 3). By contrast, Δ_f_ was similar in tob(Wt), tob(bid), and tob(flo) because of their similar Rubisco *S*_c/o_ values (Table 3). Together, Δ_e_ and Δ_f_ are expected to account for 10–18% of the carbon isotope discrimination signal in tob(Rr) and tob(L335V) compared to 6% for tob(bid) and tob(flo).

The measured Δ values are shown with respect to *C*_i_/*C*_a_ (Fig. 3). Theoretical lines are shown which assume infinite mesophyll conductance and ignore the influence of Δ_e_ and Δ_f_. Taking the Δ_e_ and Δ_f_ fractionations and mesophyll conductance into account, this study found that estimates of *b* for tob(Rr) and tob(L335V) were significantly less than the 29‰ assumed for tob(wt) (Table 3). In contrast, there was no significant difference in the *b*-values of tob(Wt), tob(bid), and tob(flo).

## Discussion

Tobacco is established as a model species for investigations into photosynthetic metabolism as it is readily transformable via nuclear and transplastomic techniques (Rodermel *et al.*, 1988; Quick *et al.*, 1991; Hudson *et al.*, 1992; Whitney *et al.*, 1999; Maliga, 2002). This study group have extensively characterized gas exchange and carbon isotope discrimination properties in this species (Evans *et al.*, 1986, 1994; Yamori *et al.*, 2010; Tazoe *et al.*, 2011; Evans and von Caemmerer, 2013). While knowing the Rubisco discrimination factor (*b*) is pivotal for fully understanding plant carbon metabolism and the impact of photosynthesis on atmospheric carbon isotope signatures (Suits *et al.*, 2005; Tcherkez *et al.*, 2011), little is known about variation in *b* as it is difficult to measure using existing *in vitro* methods (McNevin *et al.*, 2006, 2007). Tuneable diode laser absorption spectroscopy allows rapid measurements of Δ to be made concurrently with photosynthetic gas exchange. The present study used this technique to estimate *b in vivo* in a number of transplastomic tobacco genotypes. While the technique is rapid, it relies on understanding the contribution that CO_2_ diffusion and respiratory metabolism have on photosynthetic carbon isotope discrimination (equations 2 to 7). The impact of respiratory and photorespiratory fractionation was minimized by making measurements under high light and low *p*O_2_ (Table 3). Differences in δ^13^C values of the source and measuring CO_2_ also influence Δ_e_, but on average did not vary with genotype.

CO_2_ diffusion has the greatest impact on the interpretation. Lower CO_2_-assimilation rates in transplastomic tobacco genotypes compared to wild type were not accompanied by proportional reductions in stomatal conductance and this led to greater ratios of intercellular to ambient CO_2_ (*C*_i_/*C*_a_) that increased discrimination (Figs 2 and 3). Similarly, lower CO_2_-assimilation rates reduced the draw down in *p*CO_2_ from intercellular airspace to the chloroplasts which would reduce the effect of mesophyll conductance on the isotope signal. Previous measurements of transgenic tobacco with reduced amounts of Rubisco were found to have mesophyll conductances about 20–25% less than that of wild-type leaves grown under the same conditions of irradiance, temperature, and ambient CO_2_ (Evans *et al.*, 1994). When grown under elevated CO_2_, as in the present case, anti-*Rbc*S plants are indistinguishable from wild type in terms of size. Consequently, under these conditions, their mesophyll conductance would be expected to be similar. Mesophyll conductance is influenced by growth irradiance between 0.2 and 0.5mol m^2^ s^–1^ bar^–1^, having been observed for tobacco at 25 ºC (Table 2; Evans *et al.*, 1994; Yamori *et al.*, 2010; Evans and von Caemmerer, 2013). It is therefore important to measure wild-type leaves of comparable physiological age and development. Galmes *et al.* (2013) reported significantly lower *g*_m_ values calculated from chlorophyll fluorescence for tob(bid) and tob(flo) compared to wild type. Their plants were grown without CO_2_ supplementation, but under similar irradiance, photoperiod, temperature, and humidity to this study’s growth conditions. As their values for leaf dry mass per unit area, protein and Rubisco content were similar to the values measured (Table 2), the assumption that this study could use mesophyll conductance obtained from wild-type leaves needs to be kept in mind.

The lower *b*-values calculated for Rubisco from tob(L335V) and tob(Rr) determined *in vivo* from TDL measurements match those previously determined by experimentally more demanding *in vitro* methods for L335V and *R. rubrum* Rubisco (McNevin *et al.*, 2006, 2007). For both of these enzymes, the kinetic isotopic fractionation signatures provide valuable insights into variations in the Rubisco catalytic mechanism (i.e. the carbon bond-making and -cleavage reactions; Tcherkez *et al.*, 2006; McNevin *et al.*, 2007). Transplastomic modification of other L-subunit amino acids that influence the carboxylation, decarboxylation, and hydrolysis/cleavage steps of Rubisco pose a useful approach for further dissection of the mechanistic features of Rubisco catalysis. It is also feasible that examining variation in ^13^C fractionation among catalytically and phylogenetically diverse Rubiscos by a transplastomic approach, such as tob(bid) and tob(flo), may also be useful in identifying mechanisms that underlie the natural variation in Rubisco catalysis. The method used here for measuring carbon isotope discrimination by leaves during photosynthesis is experimentally robust and simple. However, it requires the generation of photoautotrophic transplastomic lines suitable for leaf gas exchange analysis. This has been challenging for some tobacco L-subunit mutations and some heterologous Rubisco isoforms where limitations in the folding and assembly requirements cannot be met by tobacco chloroplasts, thereby either restricting or preventing recombinant Rubisco biogenesis (Whitney *et al.*, 2001, 2011a; Parry *et al.*, 2013). As shown here for all four tobacco transplastomic genotypes, even if the introduced changes to Rubisco impair its synthesis [tob(bid) and tob(flo)] or compromise catalytic activity [tob(L335V) and tob(Rr)], these can be compensated by growth at elevated *p*CO_2_ to enable photoautotrophic growth to maturity in soil. Gas exchange conditions can be chosen to suit the modified catalytic properties to allow concurrent assessment of carbon isotope discrimination.

Prior assessment of the hybrid Rubiscos in tob(bid) and tob(flo) showed their catalytic properties matched those of the parental *F. bidentis* and *F. floridana* Rubiscos (Whitney *et al.*, 2011b). Catalytic properties of hybrid enzymes containing tobacco S-subunits and L-subunits from either sunflower or tomato Rubisco also reflected those of the L-subunit (Sharwood *et al.*, 2008). However, the S-subunits of Rubisco have also been shown to influence catalytic properties. Ishikawa *et al.* (2011) produced hybrid Rubisco with rice L-subunits and sorghum S-subunits which increased both *K*_c_ and *k*_ccat_ compared to wild-type rice. The *b*-values determined for Rubisco in tob(bid) and tob(flo) matched the wild type, suggesting that, despite the C_4_-like catalysis of the hybrid Rubisco in tob(bid) (i.e. increased *k*_ccat_ and *K*_c_; Table 1), there is little or no variation in the carbon isotope discrimination by these C_3_, C_3_-C_4_, and C_4_ Rubiscos *in vivo*. Whelan *et al.* (1973) measured higher average *b*-values for *Sorghum bicolor* Rubisco (33.7±6.6‰), although statistically this overlaps the range of *b*-values calculated here for tob(bid), tob(flo), and tob(Wt). Improving the rigor of inferring Rubisco mechanistic variations from Δ measurements clearly requires reliable measurement of this parameter for Rubisco isoforms with broader catalytic spectrums (Tcherkez *et al.*, 2006; McNevin *et al.*, 2007). As shown here, transplastomic introduction of C_4_-Rubiscos into tobacco plastids provides a feasible strategy to investigate the natural diversity in *b*-values for C_4_-Rubiscos that are otherwise impossible to measure by *in vivo* approaches due to the presence of their CO_2_-concentrating mechanisms. Expanding this transplastomic approach to include the catalytically distinctive Rubiscos from phylogentically diverse sources (such as non-green algae and cyanobacteria) currently remain stymied by limitations in their folding and assembly in plant chloroplasts (Kanevski *et al.*, 1999; Whitney *et al.*, 2001).
